# Methanol-related deaths: evaluation of findings in a 111-case autopsy series

**DOI:** 10.3389/fneur.2026.1863770

**Published:** 2026-09-09

**Authors:** Samet Kiyak, Muhammed Habib Önen, Ahmet Sedat Dündar, Zarema Ferik, Ramazan Kiyak, Talip Vural

**Affiliations:** 1 Department of Forensic Medicine, Faculty of Medicine, Balikesir University, Balikesir, Türkiye; 2 Council of Forensic Medicine, Bursa Forensic Medicine Group Presidency, Ministry of Justice, Bursa, Türkiye; 3 Council of Forensic Medicine, Department of Pathology, Bursa Forensic Medicine Group Presidency, Ministry of Justice, Bursa, Türkiye; 4 Department of Emergency Medicine, Faculty of Medicine, Balikesir University, Balikesir, Türkiye; 5 Department of Forensic Medicine, Faculty of Medicine, Atatürk University, Erzurum, Türkiye

**Keywords:** central nervous system, forensic autopsy, forensic pathology, methanol poisoning, mortality, neuropathology, toxicology

## Abstract

**Background:**

Methanol poisoning is a significant public health problem associated with high mortality and severe neurological damage. This study aimed to evaluate the relationship between central nervous system (CNS) findings, blood methanol levels, and hospitalization duration in fatal methanol poisoning cases.

**Materials and methods:**

A total of 111 cases with confirmed death due to methanol poisoning, autopsied between January 1, 2021 and June 30, 2025, were retrospectively analyzed. Scene investigation, clinical, toxicological, and histopathological findings were evaluated. CNS findings were analyzed in relation to methanol concentrations and duration of hospitalization. Statistical analyses were performed using IBM SPSS Statistics version 27.

**Results:**

The majority of cases occurred during the COVID-19 period (66.7%), with a marked male predominance (92.8%). Exposure most frequently occurred in domestic settings (58.6%). The mean blood methanol level was 230.2 mg/dl (range: 12–827), and levels > 100 mg/dl were detected in 59.5% of cases. Higher blood methanol levels were significantly associated with shorter hospitalization duration (*p* < 0.001). A substantial proportion of high-dose cases resulted in death within the first 48 h. CNS findings, including necrosis, hemorrhage, and severe parenchymal softening, were significantly more frequent in cases with longer durations of hospitalization (*p* < 0.001).

**Conclusion:**

Neuropathological findings were more frequently observed in cases with longer hospitalization durations. In high-dose methanol intoxication, rapid death may prevent the development of detectable structural CNS lesions; therefore, the absence of CNS pathology does not exclude fatal methanol intoxication. Furthermore, postmortem methanol levels may be reduced by medical treatment, emphasizing the need for a comprehensive forensic evaluation integrating toxicological, clinical, and treatment data.

## 1 Introduction

Methanol (CH_3_OH), also known as wood alcohol, is a colorless, volatile, flammable liquid with properties similar to ethanol. Methanol poisoning is a significant public health problem characterized by high mortality and severe neurological sequelae. It may occur following exposure to industrial solvents, antifreeze, and cleaning products, as well as the consumption of illicit or counterfeit alcoholic beverages. Periodic outbreaks of methanol poisoning, often resulting in fatal outcomes, have also been reported in Turkey, particularly due to the consumption of low-cost, illegally produced alcoholic beverages (1–3). Methanol is rapidly and easily absorbed through the gastrointestinal tract and is metabolized in the liver by alcohol dehydrogenase to formaldehyde. Formaldehyde is subsequently oxidized by aldehyde dehydrogenase to formic acid, a highly toxic metabolite. The toxic effects of methanol are primarily mediated by its metabolites, particularly formic acid (4, 5).

CNS involvement in methanol poisoning is of critical importance for both clinical diagnosis and forensic evaluation. In the early stages, nonspecific symptoms such as headache, dizziness, confusion, ataxia, and somnolence are commonly observed. As the condition progresses and metabolic acidosis deepens, patients may develop impaired consciousness, seizures, coma, and respiratory failure. From a neuropathological perspective, selective involvement of the basal ganglia, particularly the putamen, is characteristic. Imaging and autopsy findings frequently reveal necrosis, hemorrhagic changes, and diffuse cerebral edema. In addition, visual disturbances and vision loss resulting from optic nerve and retinal damage serve as important clinical clues that help distinguish methanol poisoning from other toxic alcohol exposures. In severe cases, increased intracranial pressure, brain herniation, and secondary hypoxic–ischemic brain injury may contribute to the mechanism of death (6–10).

In this study, a total of 111 autopsied cases with confirmed death due to methanol poisoning were evaluated. Scene investigation findings and sociodemographic characteristics were systematically analyzed. In addition, comprehensive toxicological analyses and detailed CNS histopathological findings were assessed together to identify patterns associated with methanol levels, duration of hospitalization, and CNS injury, and to interpret these relationships from a forensic perspective. Accordingly, this study explored whether neuropathological findings were associated with hospitalization duration and blood methanol levels, and whether rapid death in cases with high methanol concentrations might limit the development of detectable structural CNS lesions.

## 2 Materials and methods

### 2.1 Ethical approval

Ethical approval for this study was obtained from the Health Research Ethics Committee of Balikesir University Faculty of Medicine under Decision No. 2025/10-4, dated 18 November 2025. In addition, permission for the use of forensic case data was granted by the Education and Scientific Research Commission of the Council of Forensic Medicine, Ministry of Justice, Republic of Turkey (Decision No: 21589509/2026/27; Date: 10 February 2026).

### 2.2 Case selection, data collection, and grouping

**Figure 1:**
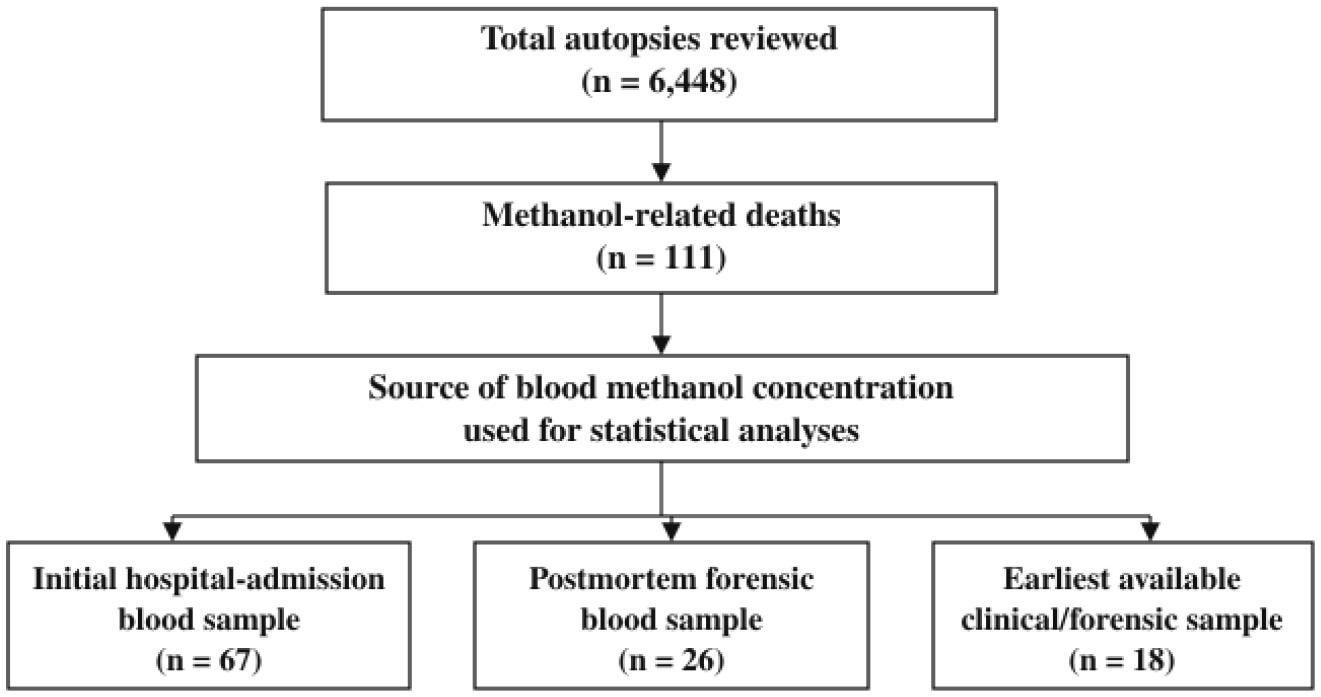
Flowchart of case selection and source of blood methanol concentrations used for statistical analyses.

The records of 6,448 cases autopsied at the Bursa Forensic Medicine Group Presidency of the Council of Forensic Medicine between January 1, 2021 and June 30, 2025 were retrospectively reviewed. Among these, 111 cases in which death was determined to be due to methanol poisoning were included in the study. Data for the included cases were obtained from institutional archives and the National Judiciary Informatics System (UYAP). The study workflow and case classification are presented in Figure 1.

Cases were identified through a comprehensive clinico-forensic evaluation integrating clinical history, toxicological findings, treatment records, autopsy findings, and judicial investigation data. For statistical analyses, the highest documented blood methanol concentration available for each case was used. This value was obtained from the initial hospital-admission blood sample (*n* = 67), the postmortem forensic blood sample (*n* = 26), or the earliest available clinical or forensic blood sample when a standardized pretreatment admission sample was unavailable (*n* = 18).

Within the scope of the study, scene investigation findings, sociodemographic characteristics, clinical records of hospitalized cases (including available documentation on patient evaluation, treatment, laboratory investigations, intensive care follow-up, and the in-hospital clinical course), toxicological analysis results, autopsy findings, histopathological examination reports, and causes of death were systematically evaluated.

During the study period, the COVID-19 pandemic affected populations worldwide, and various social restriction measures were implemented in Turkey between January 2021 and May 2022 to ensure social isolation. Accordingly, this interval was defined as the COVID period. The subsequent period, from June 2022 to June 2025, was defined as the post-COVID period. Cases were divided into two main groups according to the location of death: “scene deaths” and “hospital deaths.” Scene deaths were defined as cases in which death occurred before hospital admission, whereas hospital deaths were defined as cases in which death occurred after admission to a healthcare facility. Hospital deaths comprised cases that died after receiving medical evaluation and/or treatment following hospital admission.

Methanol concentrations and the distribution of CNS histopathological findings were compared between the scene death and hospital death groups. In addition, the association between hospitalization duration and morphological findings was statistically evaluated, considering hospitalization duration as a surrogate marker of the post-admission clinical course.

For descriptive analyses, age, sex, hospitalization duration, and blood methanol concentrations were categorized into predefined groups. Age categories were selected to represent clinically relevant demographic groups while ensuring adequate case distribution within each category. Hospitalization duration categories were defined to reflect clinically meaningful stages of the post-admission clinical course. Blood methanol concentration categories were established based on clinically and forensically relevant concentration ranges to facilitate descriptive comparisons among study groups.

Sources of methanol exposure were classified into the following categories based on forensic practice in Turkey, law enforcement reports, and the dynamics of methanol-related mass poisoning events reported between 2020 and 2022: unknown, cologne/methylated spirits, homemade alcoholic beverages, and counterfeit/illicit alcoholic beverages. Homemade alcoholic beverages were defined as products produced by individuals, particularly during the pandemic period due to restrictions and increased costs, and prone to methanol contamination as a result of insufficient technical knowledge (distillation errors). Counterfeit/illicit alcoholic beverages were defined as products in which legal packaging and labeling are imitated, but which contain high levels of industrial methanol instead of ethanol to reduce production costs and are distributed through illegal channels.

Exposure location was classified into four categories: home, hospital, workplace, and others. The “others” category included locations such as abandoned buildings, containers, apartment entrances, and public streets.

Information regarding “alcohol dependence” and “history of addiction treatment” in the cases was determined based on the decedents' past medical records (e-Nabiz system, hospital information systems, and addiction treatment center records), statements from relatives and witnesses in forensic investigation files, and scene investigation reports prepared by law enforcement authorities. These data were recorded to characterize chronic alcohol use patterns prior to methanol exposure. Only cases with documented and verifiable histories were classified as having a “history of alcohol dependence.”

### 2.3 Autopsy procedure and histopathological examination protocol

In accordance with medico-legal autopsy practices in Turkey, autopsies were performed by forensic medicine specialists at our center, and histopathological examinations were conducted by forensic pathologists. During the autopsy, the brain, cerebellum, brainstem, coronary arteries, heart, spinal cord, both lungs, liver, both kidneys, pancreas, and spleen were macroscopically examined. Following macroscopic evaluation, appropriate tissue samples were collected for histopathological analysis.

For microscopic examination, routine brain sampling included the frontal lobe gray–white matter junction, pericallosal white matter surrounding the corpus callosum, the corpus striatum (including the basal ganglia region), hippocampus, cerebellum, and brainstem, in accordance with the routine central nervous system sampling protocol used in forensic autopsy practice. Accordingly, the putamen, caudate nucleus, and globus pallidus were represented within the routinely sampled basal ganglia sections. Additional tissue samples were obtained whenever macroscopic findings required further neuropathological evaluation. Optic nerve sampling was not performed routinely but was obtained selectively in cases with documented visual symptoms before death and/or when the forensic physician considered further evaluation of the visual pathway to be necessary based on the individual findings of the case. The collected tissues were fixed in 10% formalin, processed, and embedded in paraffin blocks according to standard histological procedures.

Sections were stained with hematoxylin and eosin (*H* & *E*) and examined under light microscopy at magnifications of 40 × and 100 × . Histopathological findings of the brain, cerebellum, brainstem, liver, and other organs were evaluated in detail.

### 2.4 Toxicological analyses

During autopsy, samples from organs and body fluids (blood, vitreous humor, urine/bladder irrigation fluid, and bile) were routinely collected. Within the scope of the study, toxicological findings obtained during the medico-legal investigation, including both antemortem and postmortem analyses when available, were evaluated.

Toxicological analyses were performed in the forensic toxicology laboratory of the Council of Forensic Medicine in accordance with the institutional quality management procedures. Antemortem and postmortem blood samples intended for alcohol analysis were collected in sodium fluoride–containing tubes and stored at 4 °C until analysis to minimize the loss of volatile compounds and postmortem production of ethanol. Methanol was identified and quantified using validated headspace gas chromatography with flame ionization detection (HS–GC–FID; Clarus 580, PerkinElmer, USA), whereas gas chromatography–mass spectrometry (GC–MS; Clarus 680/Clarus SQ 8T, PerkinElmer, USA) was used for confirmatory analysis and qualitative determination of formic acid. n-Propanol was used as the internal standard for quantitative alcohol analysis. Quantitative measurements were performed using certified reference materials and multipoint calibration, and all analytical procedures were carried out in accordance with the laboratory's internal quality assurance and validation protocols. The limit of detection (LOD) and the limit of quantification (LOQ) for methanol were 3 mg/dl and 4 mg/dl, respectively. Formic acid was evaluated qualitatively (positive/negative) as part of the routine forensic toxicological examination; quantitative formic acid measurements were not available for all cases included in this retrospective study.

The presence of cases with methanol concentrations below fatal levels (12–19.9 mg/dl) in the study group was carefully evaluated for diagnostic reliability. Low methanol concentrations were observed predominantly among hospitalized cases that had received medical care before forensic evaluation. Because detailed treatment records were not consistently available, the potential influence of therapeutic interventions on postmortem methanol concentrations could not be determined. Therefore, low postmortem methanol concentrations should be interpreted in the context of clinical history, available medical records, and the interval between exposure and specimen collection.

For the forensic diagnosis of methanol poisoning, a comprehensive clinico-forensic approach was applied by integrating toxicological findings with clinical records, treatment history, autopsy findings, and investigative information. In cases with multiple toxicological measurements, the highest documented blood methanol concentration obtained from either the antemortem sample or the postmortem forensic sample was used for statistical analyses. This approach was adopted to better reflect the highest documented methanol exposure while reducing the potential influence of temporal changes in methanol concentrations during clinical management. The case selection process and the sources of blood methanol concentrations used for statistical analyses are illustrated in Figure 1. The measured methanol concentrations were therefore interpreted as routinely available forensic toxicological data rather than as standardized pharmacokinetic measurements obtained at uniform intervals after exposure.

### 2.5 Statistical analysis

Because the exact interval between methanol exposure and death could not be reliably determined in most cases, hospitalization duration was used as a surrogate marker of the post-admission clinical course, and all corresponding analyses and interpretations were made within this context.

Accordingly, all statistical analyses were performed using the final medico-legally confirmed study cohort, as the primary objective of the study was to characterize the forensic and neuropathological features of confirmed methanol poisoning deaths rather than to evaluate diagnostic performance.

Data from methanol poisoning cases were entered into an Excel database and analyzed using IBM SPSS Statistics version 27.0 (IBM Corp., Armonk, NY, USA). Variables were presented using descriptive statistics as number (*n*) and percentage (%). Associations between categorical variables, including location of death (hospital vs. scene), presence of CNS findings (present/absent), and methanol level categories (12–19.9, 20–50.9, 51–100, and > 100 mg/dl), were evaluated using the Pearson chi-square (χ^2^) test.

All analyses were performed with 95% confidence intervals, and *p*-values of < 0.05 were considered statistically significant. Given the descriptive nature of the study, statistical analyses were performed to evaluate associations rather than to establish causal relationships.

## 3 Results

### 3.1 Sociodemographic characteristics, scene investigation, treatment process, autopsy, and toxicological analyses

The study covered a 4.5-year period, with most cases occurring during the COVID-19 restriction period (January 2021–May 2022) (66.7%). The 50–59 age group constituted the largest proportion (43.2%). Most cases were male (92.8%), while female cases were limited (7.2%).

A notable proportion of cases lacked information regarding toxic exposure at the time of autopsy (45.0%). Among cases with available exposure data, the most frequently reported source was cologne/methylated spirits (28.8%), followed by homemade alcoholic beverages (13.5%) and counterfeit alcoholic beverages (12.6%). When exposure sources were examined descriptively according to the study period, cologne/methylated spirits exposure was documented in 23 cases during the COVID period and in 9 cases during the post-COVID period. Homemade alcoholic beverage exposure was documented in 6 cases during the COVID period and in 9 cases during the post-COVID period. Counterfeit alcoholic beverage exposure was documented in 10 cases during the COVID period and in 4 cases during the post-COVID period. A history of alcohol dependence was identified in a limited number of cases (10.8%). The majority of cases died after hospital admission (76.6%), while the remaining cases died at the scene (23.4%). Among the 26 scene deaths, 22 occurred in domestic settings, whereas the remaining four occurred in open areas, including streets, roadsides, open fields, and agricultural areas. Exposure most occurred in domestic settings (58.6%), and a considerable proportion of deaths also took place at home (19.8%) (Table 1).

**Table 1:** Sociodemographic, clinical, toxicological, and scene characteristics of the cases.

Characteristic	Category	*n*	%
Study period	COVID-19 period^*^	74	66.7
	Post-COVID period^**^	37	33.3
Age (years)	< 35	7	6.3
	35–49	38	34.2
	50–59	48	43.2
	> 59	18	16.2
Sex	Male	103	92.8
	Female	8	7.2
Exposure source	Unknown	50	45.0
	Cologne/methylated spirits	32	28.8
	Homemade alcoholic beverages	15	13.5
	Counterfeit alcoholic beverages	14	12.6
History of alcohol dependence	Present	12	10.8
	Absent	99	89.2
Place of death	Hospital	85	76.6
	Scene	26	23.4
Treatment status	No	26	23.4
	Yes	85	76.6
Ethanol therapy	No treatment history	26	23.4
	Ethanol administered	46	41.5
	Treatment given, ethanol administration unknown	39	35.1
ICU length of stay	No ICU stay	26	23.4
	1–2 days	51	46.0
	3–7 days	21	18.9
	8–14 days	6	5.4
	≥ 15 days	7	6.3
Methanol detection at autopsy	Negative	19	17.1
	Positive	92	82.9
Formic acid detection at autopsy	Negative	82	73.9
	Positive	29	26.1
Blood methanol level (mg/dl)	12–19.9	18	16.2
	20–50.9	6	5.4
	51–100	21	18.9
	>100	66	59.5
Other toxicological findings (blood/urine)	None detected	99	89.2
	Antidepressant drugs	8	7.2
	Narcotic/stimulant/inhalant substances (amphetamine, methamphetamine, benzoylecgonine, THC-COOH, toluene)	4	3.6

ICU, Intensive care unit. “Negative” indicates that methanol was not detected in the postmortem blood sample. This variable is independent of the Blood methanol level (mg/dl) categories, which were based on the highest documented blood methanol concentration available for statistical analyses, obtained from either an antemortem or a postmortem blood sample. Therefore, postmortem methanol-negative cases may still be included in the 12–19.9 mg/dl blood methanol category if methanol was detected in an antemortem blood sample. The diagnosis of fatal methanol poisoning was established through an integrated clinico-forensic evaluation. Information regarding fomepizole administration and hemodialysis was not systematically available or sufficiently reliable in the retrospective medico-legal records; therefore, these variables were not included in Table 1 or subsequent statistical analyses.

^*^COVID-19 period: January 2021 to May 2022, during which national restrictions were implemented in Turkey.

^**^Post-COVID period: June 2022 to June 2025.

Other toxicological findings (blood/urine): in some cases, multiple toxic substances were detected.

The majority of cases received hospital treatment (76.6%), and ethanol therapy was administered in 41.5% of these cases. In 35.1% of cases, information regarding the administration of ethanol therapy could not be obtained. A considerable proportion of patients admitted to the intensive care unit died within 1–2 days (46%).

Toxicological analyses revealed the presence of methanol in 82.9% of cases. Among methanol-positive cases, the mean blood methanol level was 230.2 mg/dl (range: 12–827 mg/dl). The > 100 mg/dl category accounted for the largest proportion of cases (59.5%). Cases that died at the scene were more frequently represented in the higher methanol concentration categories than hospital deaths (*p* = 0.003). Methanol was not detected in postmortem blood samples in 17.1% of cases. It was determined that the cases in which methanol was not detected had particularly prolonged hospital stays in district state hospitals, where methanol analysis could not be performed in the laboratories due to technical limitations. Qualitative formic acid analysis was positive in 29 cases (26.1%) and negative in 82 cases (73.9%). Other toxicological substances were largely negative (89.2%), with only a limited number of cases showing the presence of antidepressant drugs (7.2%) or narcotic/stimulant/inhalant substances (3.6%) (Table 1).

### 3.2 Histopathological examination at autopsy

**Figure 2:**
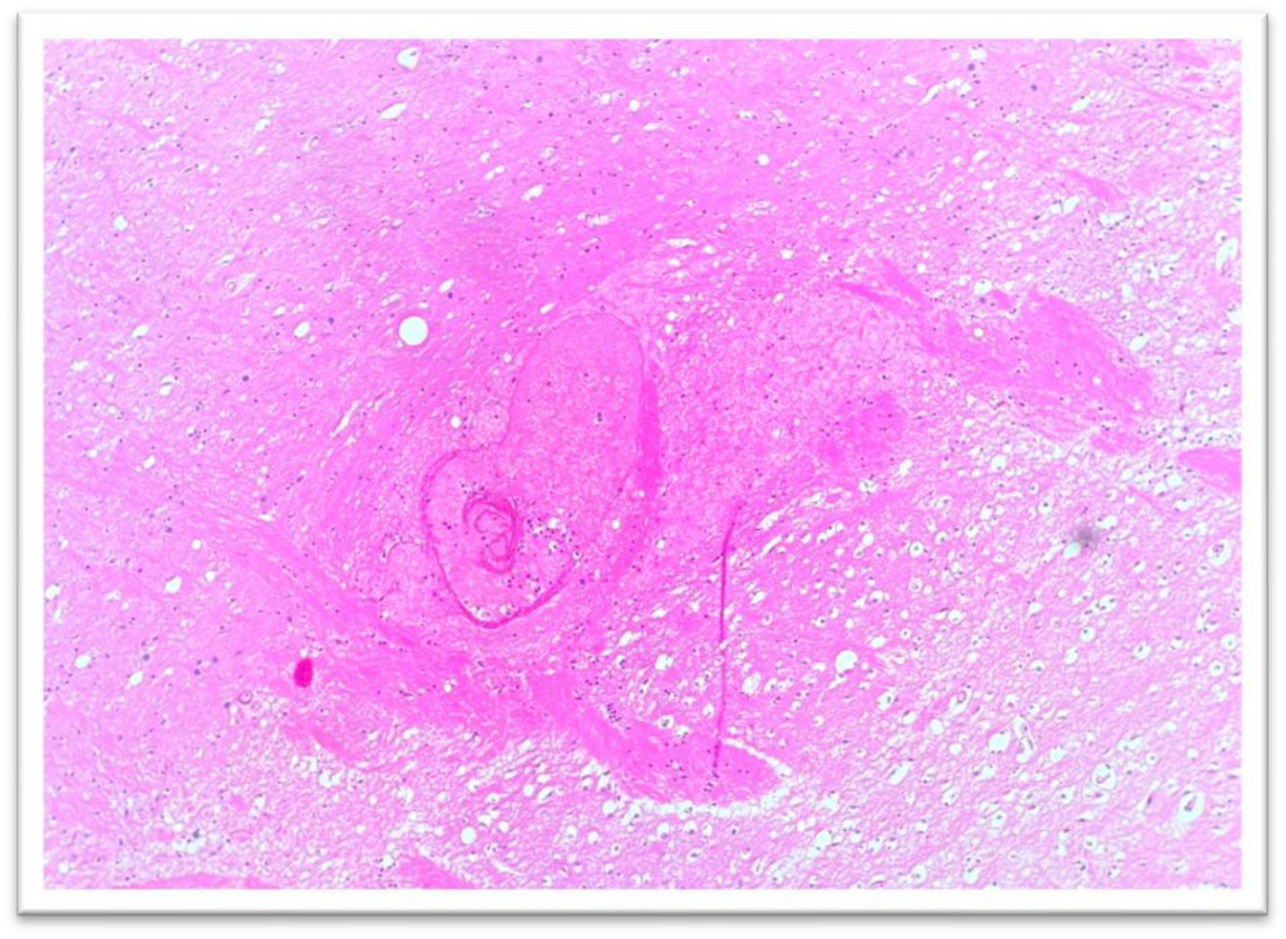
Brain histopathology in methanol poisoning showing focal hemorrhage and necrosis in glial tissue (*H* & *E*, × 10).

**Figure 3:**
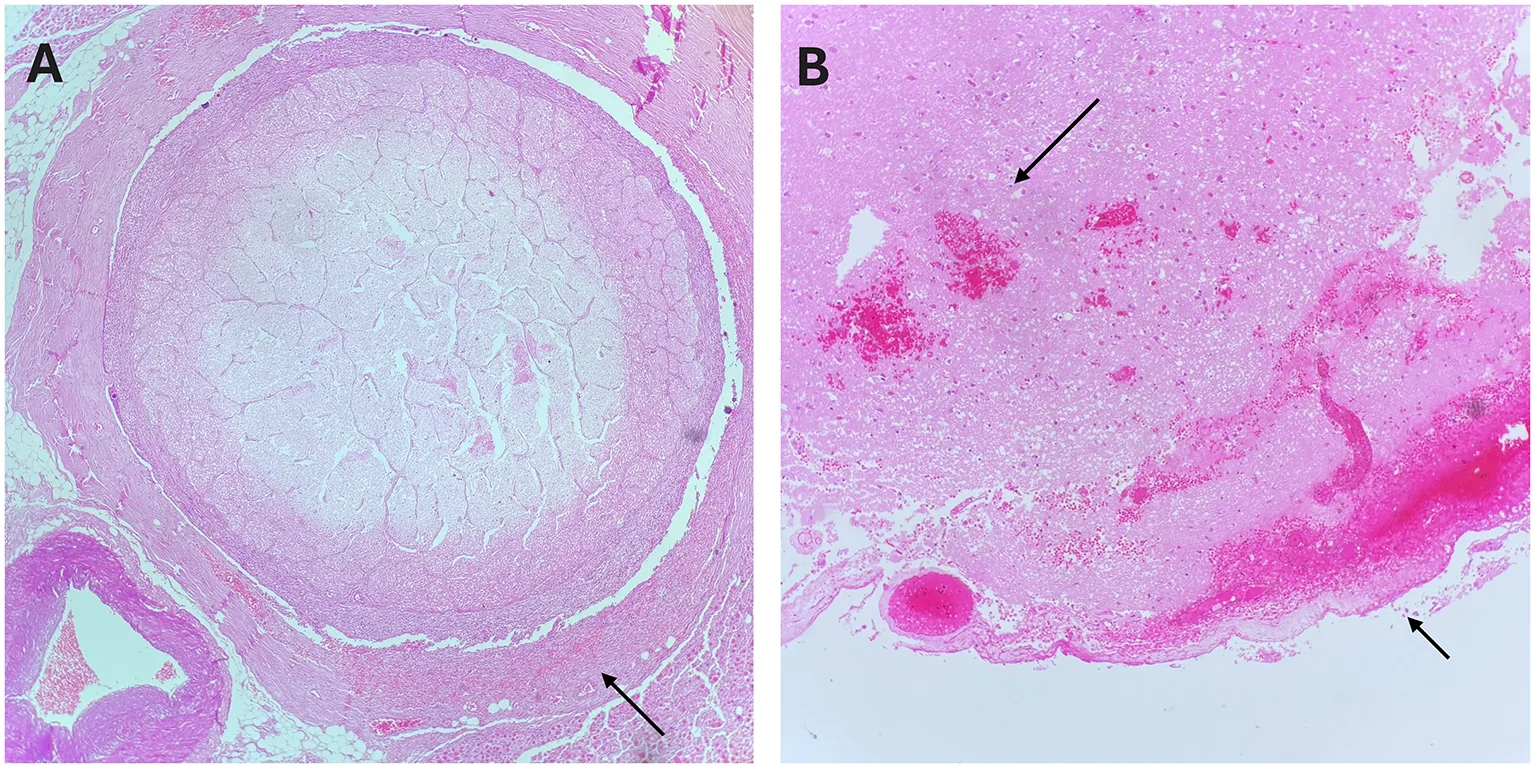
Brain and optic nerve histopathology in methanol poisoning showing hemorrhage within the optic nerve sheath (A, arrow) and subarachnoid (B, small arrow) and subcortical hemorrhages (B, large arrow) (*H* & *E*, × 4).

Histopathological examination revealed significant CNS findings in 51.4% of cases. Evaluation of CNS findings demonstrated that the most common pathological changes were necrosis and hemorrhage. Necrosis without specified localization in the brain was identified in 21.6% of cases, while hemorrhage without necrosis was observed in 16.2%. Necrosis involving the brainstem and cerebellum was detected in 9.9% of cases, and diffuse hypoxia/old lesion findings were identified in 3.6%. Overall, necrosis was present in 31.5% of cases (Figure 2), and hemorrhage was observed in 39.6% (Figure 3) (Table 2). Analysis by hospitalization duration demonstrated that both CNS hemorrhage and CNS necrosis were more frequently observed in cases with longer hospitalization durations (*p* < 0.001 for both comparisons).

**Table 2:** Distribution of CNS, liver, and optic nerve histopathological findings in methanol poisoning cases.

Findings	Category	*n*	%
Significant CNS histopathological findings	No significant findings	54	48.6
	Hemorrhage present without necrosis	18	16.2
	Cerebral necrosis (with or without hemorrhage)	24	21.6
	Necrosis in the brainstem and cerebellum (with or without hemorrhage)	11	9.9
	Diffuse hypoxia and old lesions	4	3.6
Overall cns necrosis	Present	35	31.5
Overall CNS hemorrhage	Present	44	39.6
Secondary CNS findings	CNS edema	66	59.5
	CNS congestion	83	74.8
Macroscopic brain consistency	No softening	72	64.9
	Mild softening	15	13.5
	Severe softening	24	21.6
Liver histopathology	No steatosis	19	17.1
	Hepatosteatosis without cirrhosis	82	73.9
	Hepatosteatosis with cirrhosis	8	7.2
	Not evaluated	2	1.8
Optic nerve histopathology	Not evaluated	100	90.1
	Evaluated	11	9.9

CNS congestion was considered a nonspecific secondary autopsy finding and was therefore not classified as a significant methanol-related histopathological lesion.

Evaluation of secondary CNS histopathological findings showed that congestion was the most common finding (74.8%), followed by edema (59.5%).

**Figure 4:**
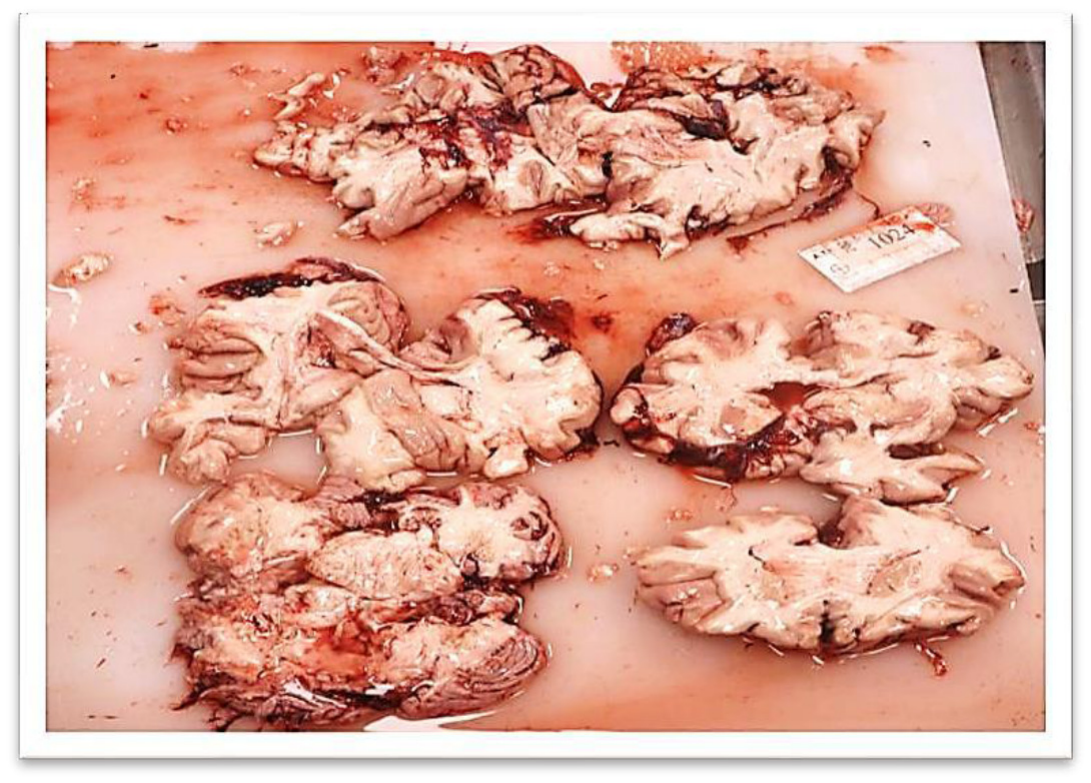
Macroscopic brain sections in methanol poisoning demonstrating diffuse parenchymal softening with associated hemorrhagic areas.

Macroscopic examination revealed that softening was absent in the majority of cases (64.9%), whereas severe softening was identified in 21.6% of cases (Figure 4).

**Figure 5:**
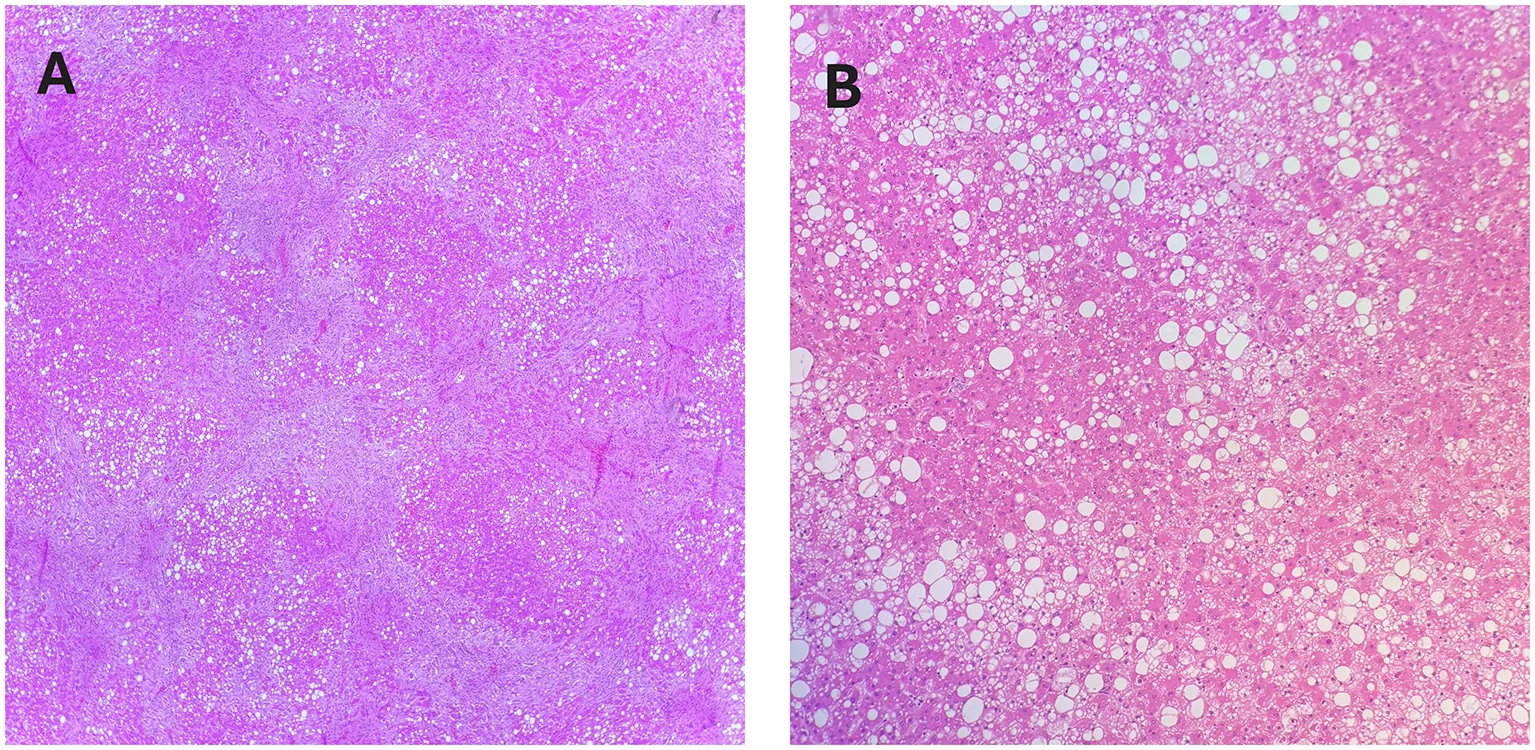
Liver histopathology in methanol poisoning showing cirrhotic architecture **(A)** and diffuse microvesicular and macrovesicular steatosis in hepatocytes **(B)** (*H* & *E*, × 10).

In liver histopathology, hepatosteatosis was identified in 90 cases (81.1%), including 82 cases (73.9%) without cirrhosis and 8 cases (7.2%) with cirrhosis (Figure 5).

Optic nerve histopathology was not evaluated in the majority of cases (90.1%). Among the limited number of evaluable cases, findings were predominantly normal (7.2%) (Table 2).

In cases with blood methanol levels < 100 mg/dl, CNS hemorrhage and CNS necrosis were observed at significantly higher rates than in cases with blood methanol levels > 100 mg/dl (*p* = 0.015 and *p* = 0.045, respectively). In contrast, no statistically significant differences were found between the methanol level groups for CNS edema or macroscopic consistency changes (*p* = 0.766 and *p* = 0.213, respectively) (Tables 3, 4).

**Table 3:** Distribution of histopathological findings and duration of hospitalization according to blood methanol levels.

CNS histopathological findings	Blood methanol level (mg/dl)				*n*	%
	<20	20–50	51–100	>100		
No significant findings	4	3	8	39	54	48.6
Hemorrhage present without necrosis	4	1	5	8	18	16.2
Brain necrosis (with or without hemorrhage, localization not specified)	8	0	7	9	24	21.6
Necrosis in the brainstem and cerebellum (with or without hemorrhage)	2	2	0	7	11	9.9
Diffuse hypoxia and old lesions	0	0	1	3	4	3.6
**CNS edema**						
Present	9	3	14	40	66	59.5
Absent	9	3	7	26	45	40.5
**CNS congestion**						
Present	15	5	16	47	83	74.8
Absent	3	1	5	19	28	25.2
**CNS hemorrhage**						
Present	11	1	12	20	44	39.6
Absent	7	5	9	46	67	60.4
**CNS necrosis**						
Present	10	2	7	16	35	31.5
Absent	8	4	14	50	76	68.5
**Macroscopic brain consistency**						
No softening	5	6	14	47	72	64.9
Softened	3	0	4	8	15	13.5
Severely softened	10	0	3	11	24	21.6
**Liver histopathology**						
Not evaluated	0	0	1	1	2	1.8
No steatosis	4	1	5	9	19	17.1
Hepatosteatosis without cirrhosis	14	4	12	52	82	73.9
Hepatosteatosis with cirrhosis	0	1	3	4	8	7.2
**Optic nerve histopathology**						
Not evaluated	17	6	18	59	100	90.1
Normal	1	0	3	4	8	7.2
Congestion	0	0	0	3	3	2.7
**Duration of hospitalization**						
0 days	1	1	2	24	28	25.2
1–2 days	5	2	8	34	49	44.1
3–7 days	8	2	6	5	21	18.9
8–14 days	2	1	1	2	6	5.4
≥15 days	2	0	4	1	7	6.3
**Total**	**18**	**6**	**21**	**66**	**111**	**100**

**Table 4:** Statistical analysis of histopathological findings and duration of hospitalization according to blood methanol levels.

CNS histopathological findings	Blood methanol level (mg/dl)		
	≤ 100 (%)	>100 (%)	Test (chi-square)
**CNS edema**			
Present	26 (57.8)	40 (60.6)	*p* = 0.766
Absent	19 (42.2)	26 (39.4)	
Total	45 (100)	66 (100)	
**CNS hemorrhage**			
Present	24 (53.3)	20 (30.3)	*p* = 0.015
Absent	21 (46.7)	46 (69.7)	
Total	45 (100)	66 (100)	
**CNS necrosis**			
Present	19 (42.2)	16 (24.2)	*p* = 0.045
Absent	26 (57.8)	50 (75.8)	
Total	45 (100)	66 (100)	
**Macroscopic brain consistency**			
No softening	25 (55.6)	47 (71.2)	*p* = 0.213
Softened	7 (15.6)	8 (12.1)	
Severely softened	13 (28.8)	11 (16.7)	
Total	45 (100)	66 (100)	
**Duration of hospitalization**			
0 days	4 (8.9)	24 (36.4)	*p* < 0.001
1–2 days	15 (33.3)	34 (51.5)	
3–7 days	16 (35.6)	5 (7.6)	
≥ 8 days	10 (22.2)	3 (4.5)	
Total	45 (100)	66 (100)	

A statistically significant association was identified between blood methanol level categories and hospitalization duration (*p* < 0.001). A large proportion of cases with high methanol levels had no hospitalization or a short duration of hospitalization.

Hospitalization duration was significantly associated with the presence of CNS hemorrhage and necrosis (both *p* < 0.001). Hemorrhage was observed in 3.6% of cases with 0 days of hospitalization and increased to 42.9, 66.7, and 61.5% among cases hospitalized for 1–2 days, 3–7 days, and ≥ 8 days, respectively. Similarly, the frequency of necrosis increased from 7.1 to 24.5%, 61.9%, and 61.5% across the corresponding hospitalization categories (*p* < 0.001). In contrast, no statistically significant association was found between CNS edema and hospitalization duration (*p* = 0.734) (Tables 5, 6).

**Table 5:** CNS histopathological findings according to duration of hospitalization.

Findings	0 days	1–2 days	3–7 days	8–14 days	≥15 days	*n*	%
15.5-7.4,14175.3mm**CNS histopathological findings**							
No significant findings	25	24	3	2	0	54	48.6
Hemorrhage present without necrosis	0	11	5	1	1	18	16.2
Brain necrosis (with or without hemorrhage, localization not specified)	1	8	8	3	4	24	21.6
Necrosis in the brainstem and cerebellum (with or without hemorrhage)	1	4	5	0	1	11	9.9
Diffuse hypoxia and old lesions	1	2	0	0	1	4	3.6
Total	28	49	21	6	7	111	100
**CNS edema**							
Present	18	29	13	4	2	66	59.5
Absent	10	20	8	2	5	45	40.5
Total	28	49	21	6	7	111	100
**CNS congestion**							
Present	24	35	16	4	4	83	74.8
Absent	4	14	5	2	3	28	25.2
Total	28	49	21	6	7	111	100
**CNS hemorrhage**							
Present	1	21	14	3	5	44	39.6
Absent	27	28	7	3	2	67	60.4
Total	28	49	21	6	7	111	100
**CNS necrosis**							
Present	2	12	13	3	5	35	31.5
Absent	26	37	8	3	2	76	68.5
Total	28	49	21	6	7	111	100
**Macroscopic brain consistency**							
No softening	23	39	6	2	2	72	64.9
Softened	5	3	6	0	1	15	13.5
Severely softened	0	7	9	4	4	24	21.6
Total	28	49	21	6	7	111	100

**Table 6:** Statistical analysis of CNS histopathological findings according to duration of hospitalization.

CNS histopathological findings	0 days (%)	1–2 days (%)	3–7 days (%)	≥8 days (%)	Test (chi-square)
**CNS edema**					
Present	18 (64.3)	29 (59.2)	13 (61.9)	6 (46.2)	*p* = 0.734
Absent	10 (35.7)	20 (40.8)	8 (38.1)	7 (53.8)	
Total	28 (100)	49 (100)	21 (100)	13 (100)	
**CNS hemorrhage**					
Present	1 (3.6)	21 (42.9)	14 (66.7)	8 (61.5)	*p* < 0.001
Absent	27 (96.4)	28 (57.1)	7 (33.3)	5 (38.5)	
Total	28 (100)	49 (100)	21 (100)	13 (100)	
**CNS necrosis**					
Present	2 (7.1)	12 (24.5)	13 (61.9)	8 (61.5)	*p* < 0.001
Absent	26 (92.9)	37 (75.5)	8 (38.1)	5 (38.5)	
Total	28 (100)	49 (100)	21 (100)	13 (100)	

## 4 Discussion

Methanol toxicity has long been extensively studied because of its severe neurotoxic effects in humans (3, 9–12). The main strength of this study lies in its relatively large autopsy-based series of methanol-related deaths (*n* = 111) and the systematic evaluation of sociodemographic characteristics together with scene investigation findings. The study period encompassed both the COVID-19 restriction period and the subsequent post-pandemic period, allowing the temporal distribution of cases to be described. Notably, 74 of the 111 cases (66.7%) occurred during the COVID period, despite the shorter duration of this interval compared with the post-COVID period. Previous studies have suggested that pandemic-related restrictions, social isolation, economic difficulties, and changes in access to alcoholic products may have influenced patterns of methanol exposure and healthcare utilization (13, 14). These factors may have contributed to increased reliance on unregulated or illicit alcoholic products; however, given the retrospective and descriptive design of the present study, the observed temporal differences should be interpreted as associations and do not establish a causal relationship.

In our study, the predominance of the 50–59 age group (43.2%) and the marked male predominance (92.8%) are consistent with previous epidemiological studies on the demographic distribution of methanol-related mortality (7, 9, 11, 15). This pattern may be associated with high-risk consumption behaviors and sociocultural differences in exposure. In the context of Turkey, the fact that the procurement and consumption of illicit alcohol often occur within male-dominated social networks may represent an important factor explaining this pronounced gender distribution.

On the other hand, the high proportion of missing exposure source data (45.0%) represents a significant limitation to data completeness in the retrospective evaluation of fatal cases. Nevertheless, among cases with available exposure source information, the predominance of cologne/methylated spirits use (28.8%), followed by homemade and counterfeit/illicit alcoholic beverages, suggests that both increased reliance on alcohol-based products and the consumption of unregulated alcoholic beverages may have contributed to methanol exposure during the pandemic period.

The relatively low prevalence of alcohol dependence in the medical history (10.8%) suggests that methanol exposure is not limited to individuals with known alcohol use disorders. This finding indicates that methanol poisoning can result in fatal outcomes even among “unexpected” user groups. It further supports the notion that prevention strategies should not be restricted solely to the framework of chronic alcohol dependence but should also incorporate structural approaches such as regulatory control mechanisms, product safety, proper labeling, and standardized documentation of scene investigation findings.

In our study, the majority of cases died during hospital treatment (76.6%), whereas a smaller proportion died at the scene. Domestic settings were the most frequently documented exposure location (58.6%), and 22 scene deaths (19.8% of all cases) occurred at home. Taken together, these findings identify domestic environments as an important setting for fatal methanol exposure in this cohort. Such a pattern may reflect the consumption of unregistered or counterfeit alcoholic beverages in domestic environments or accidental exposure to methanol-containing household products. These observations underscore the importance of awareness-raising and prevention efforts addressing domestic methanol exposure.

Because the initial clinical presentation of methanol poisoning may be nonspecific, early recognition and prompt medical evaluation remain important (10, 16). In addition, careful documentation and collection of suspicious substances at the scene may facilitate subsequent forensic investigations.

Evaluation of treatment records in hospitalized cases revealed notable findings regarding clinical management and data completeness. While ethanol therapy was administered in 41.5% of cases, information regarding ethanol administration was unavailable in 35.1% of cases, highlighting the incomplete treatment documentation frequently encountered in retrospective forensic-toxicological studies. Since inhibition of alcohol dehydrogenase with ethanol or fomepizole represents one of the main antidotal treatment strategies in methanol poisoning, and treatment timing plays a decisive role in determining prognosis (10, 16, 17), standardized documentation of antidotal treatment protocols and renal replacement therapies is essential for both clinical quality improvement and the reliability of forensic evaluations.

The finding that a substantial proportion of cases died within 1–2 days after admission to the intensive care unit (46%) is consistent with the high blood methanol concentrations observed in our study and reflects the severe clinical course of methanol intoxication. Methanol toxicity is primarily driven by the accumulation of formic acid, leading to metabolic acidosis and neuro-ocular injury, and in severe cases, may rapidly progress to multi-organ dysfunction and death (10, 16, 17).

Toxicological analyses demonstrated the presence of methanol in 82.9% of cases. In the remaining 17.1% of cases in which methanol could not be detected, toxicological analyses could not be performed at the initial healthcare facilities due to technical limitations, and the diagnosis was established based on the available clinical findings. In these cases, the absence of methanol in postmortem samples may be related to the interval between exposure and postmortem sampling. Because detailed treatment information was not consistently available, the potential influence of therapeutic interventions on postmortem methanol concentrations could not be evaluated.

In our study, the presence of cases with methanol levels 12–19.9 mg/dl indicates that postmortem toxicological analysis should not be considered a standalone “gold standard.” The lowest postmortem blood methanol concentration detected in our series was 12 mg/dl. Previous studies have shown that therapeutic interventions may influence postmortem methanol concentrations (16). Since all low-level cases in our study had received hospital treatment, the measured postmortem methanol concentrations may not accurately reflect the initial exposure level. Furthermore, because detailed treatment records were unavailable for some cases, it was not possible to distinguish specific therapeutic interventions. Therefore, postmortem toxicological findings should be interpreted in conjunction with the clinical history, duration of hospitalization, and available treatment information to avoid misinterpreting treatment-related low methanol concentrations as evidence of non-fatal exposure.

The reported lethal oral dose of methanol ranges between 30 and 240 ml, with the usual fatal dose estimated at 100–250 ml (18, 19). In our study, the mean blood methanol level among methanol-positive cases (*n* = 92) was 230.2 mg/dl (range: 12–827), and the predominance of the > 100 mg/dl category (59.5%) suggests severe toxicity, consistent with high-dose exposure in a substantial proportion of cases. Methanol levels were significantly higher in cases who died before hospital admission, suggesting an association between higher methanol concentrations and death before hospitalization. Although clinical deterioration in methanol poisoning is not solely determined by methanol concentration but is also influenced by the depth of metabolic acidosis resulting from formic acid accumulation, elevated methanol levels remain an important indicator of a severe clinical course and increased mortality risk (9–11).

Methanol concentrations below 20 mg/dl may still be clinically hazardous, as emphasized in the literature (9, 11, 20, 21). According to forensic evaluation guidelines used within the framework of the Turkish Penal Code, methanol intoxication is considered life-threatening when supported by clinical findings, particularly in cases with blood methanol concentrations ≥ 30 mg/dl or the presence of visual impairment (22). Our findings are consistent with the current medico-legal approach, emphasizing that blood methanol concentration should be interpreted in conjunction with the clinical presentation and other forensic findings rather than as an isolated criterion.

The absence of other toxicological substances in the majority of cases (89.2%) further strengthens the forensic interpretation that methanol was the primary toxic agent. The detection of antidepressants (7.2%) and narcotic/stimulant/inhalant substances (3.6%) indicates concomitant substance use in a limited number of cases. However, the potential clinical impact of these substances could not be evaluated within the scope of the present study. These findings highlight the importance of combining targeted toxicological analyses with comprehensive screening panels and thorough scene investigation in forensic toxicology practice.

In this study, significant CNS histopathological findings were detected in more than half of the cases (51.4%), demonstrating frequent neuropathological involvement in fatal methanol poisoning. Methanol toxicity is primarily mediated by its metabolite formic acid, which induces hypoxia-like cellular energy failure through inhibition of mitochondrial cytochrome c oxidase, with the CNS and visual pathways being particularly vulnerable. This mechanism provides a biological basis for the severe lesions observed in our study, including necrosis and hemorrhage (10, 16, 23).

Although higher blood methanol concentrations were associated with shorter hospitalization durations, necrosis and hemorrhage were observed more frequently in cases with longer hospitalization durations. These findings suggest that the development of structural CNS injury may depend not only on the severity of exposure but also on the time available for neuropathological changes to evolve. Necrosis with a hemorrhagic component is most commonly reported in the putamen and other basal ganglia, although diffuse hypoxic–ischemic injury may also occur in severe cases (11, 23). In our study, a considerable proportion of necrosis (21.6%) was reported in the brain without a specified anatomical localization (putamen and other basal ganglia). This suggests that global hypoxic–ischemic or widespread toxic–metabolic injury may have contributed to the observed neuropathological findings. Necrosis identified in the brainstem and cerebellum (9.9%) may reflect terminal events such as severe hypoxia, circulatory compromise, or brain herniation (9, 10, 16, 23–25). However, histopathological evaluations were performed at a general level without detailed subgrouping of specific brain nuclei, representing a limitation of the present study.

The predominance of congestion (74.8%) and edema (59.5%) among secondary histopathological findings is consistent with hypoxia, metabolic acidosis, increased capillary permeability, and terminal circulatory disturbances frequently observed in methanol poisoning. The detection of severe macroscopic softening in 21.6% of cases may suggest that, in a subset of cases, the pre-mortem process was prolonged enough for tissue-level changes to become apparent, or that severe edema and/or necrotic alterations had developed. However, it should be noted that factors such as postmortem interval and autolysis may influence the assessment of “softening” in routine autopsy practice (26, 27).

In liver histopathology, hepatosteatosis was identified in 90 cases (81.1%), including 82 cases (73.9%) without cirrhosis and 8 cases (7.2%) with cirrhosis, suggesting that these findings are likely multifactorial. In addition to chronic alcohol use, nutritional deficiencies, and metabolic risk factors, methanol-induced mitochondrial dysfunction may also have contributed to hepatic injury (9, 16, 28, 29). However, the relative contribution of these factors could not be determined within the scope of the present study.

The fact that optic nerve histopathology was not evaluated in the majority of cases (90.1%) represents an important limitation, given that optic neuropathy is one of the most characteristic targets of methanol toxicity. The predominance of normal findings in the limited number of evaluated cases (*n* = 8) is not generalizable because of the small sample size and potential sampling variability. Recent neuropathological and neuro-ophthalmological studies have demonstrated that methanol-induced optic nerve injury may occur even in the absence of extensive structural brain lesions and that the severity of visual impairment is not always proportional to the extent of detectable neuropathological changes. These findings further emphasize the importance of standardized optic nerve and retinal sampling with clinicopathological correlation in future forensic autopsy studies (10, 30–32).

In our study, the mean blood methanol level of 230.2 mg/dl was well above the fatal thresholds reported in the literature. Notably, a substantial proportion of cases with methanol levels exceeding 100 mg/dL either died before hospital admission or had short durations of hospitalization. Dikme et al. (28), in their study of methanol poisoning cases in Istanbul, reported that high initial methanol concentrations may lead to rapid cardiovascular collapse through fulminant metabolic acidosis, thereby limiting the time available for the development of structural neurological injury. These observations are consistent with our finding that hospitalization duration, rather than methanol concentration alone, was significantly associated with the development of histopathological CNS lesions. A significant association was observed between hospitalization duration and histopathological findings, including necrosis and hemorrhage. As reported by Sanci et al. (11), structural brain damage—particularly putaminal necrosis—becomes apparent after a “window period” during which the cytotoxic effects of formic acid accumulate. Similarly, in our series, necrosis and severe parenchymal softening were most frequently observed in patients who survived long enough to allow these structural changes to evolve. Therefore, the absence of histopathological findings at postmortem examination should not be interpreted as evidence against fatal methanol intoxication, particularly in patients who die shortly after severe exposure. Despite these findings, several limitations should be considered when interpreting the results of this study. The availability of clinical and treatment-related information depended on the documentation incorporated into the forensic case records. Consequently, admission ethanol concentrations, arterial blood gas parameters (pH, anion gap, osmolar gap), and information regarding specific therapeutic interventions, including fomepizole administration and hemodialysis, were not consistently available for all cases and therefore could not be reliably analyzed. In addition, the timing and source of blood sampling were heterogeneous. The methanol concentrations used in this study were derived from admission, subsequent clinical, or postmortem blood specimens, which do not necessarily represent equivalent biological time points. Furthermore, treatment, ongoing metabolism, hemodialysis, and the interval between exposure and specimen collection may have influenced the measured methanol concentrations. Therefore, concentration-based findings should be interpreted as descriptive forensic associations within this medico-legal cohort rather than as direct pharmacokinetic or causal relationships.

## 5 Conclusion

In conclusion, the development of structural central nervous system lesions, including necrosis, hemorrhage, and severe parenchymal softening, appears to be associated with longer durations of hospitalization. These findings suggest that structural brain injury is more frequently identified in cases with longer hospitalization durations. However, in cases of very high methanol exposure, death may occur before detectable structural CNS changes have developed; therefore, a normal postmortem CNS examination does not exclude fatal methanol poisoning. Furthermore, postmortem methanol levels may be reduced, particularly in hospitalized cases, due to elimination processes associated with treatment. Accordingly, forensic evaluation should adopt a comprehensive approach that integrates toxicological findings with clinical history and administered antidotal or dialysis treatments.

Especially during crisis periods such as pandemics, increased consumption of unregulated alcohol and insufficient control mechanisms may contribute to a rise in methanol-related deaths. Early diagnosis, standardized treatment protocols, and systematic documentation of scene investigation findings remain essential for reducing mortality and improving the reliability of forensic assessments.

## Data Availability

The data used in this study are derived from official forensic case records of the Council of Forensic Medicine, Ministry of Justice, Republic of Turkey. Due to legal and institutional restrictions, the data are not publicly available. They can, however, be obtained from the corresponding author upon reasonable request, subject to the necessary permissions from the relevant authorities.
